# Attitudes and Perceptions of Health Protection Measures Against the Spread of COVID-19 in Italy and Poland

**DOI:** 10.3389/fpsyg.2021.805790

**Published:** 2021-12-24

**Authors:** Liliana Lorettu, Giuseppe Mastrangelo, Joanna Stepien, Jakub Grabowski, Roberta Meloni, Davide Piu, Tomasz Michalski, Przemyslaw M. Waszak, Saverio Bellizzi, Luca Cegolon

**Affiliations:** ^1^Department of Medical, Surgical and Experimental Sciences, University of Sassari, Sassari, Italy; ^2^Department of Cardiac, Thoracic, Vascular Sciences and Public Health, University of Padua, Padua, Italy; ^3^Department of Socio-Economic Geography, Faculty of Social Sciences, University of Gdańsk, Gdańsk, Poland; ^4^Department of Developmental Psychiatry, Psychotic and Geriatric Disorders, Medical University of Gdańsk, Gdańsk, Poland; ^5^Department of Regional Development, Faculty of Social Sciences, University of Gdańsk, Gdańsk, Poland; ^6^Department of Hygiene and Epidemiology, Medical University of Gdańsk, Gdańsk, Poland; ^7^Independent Consultant, Geneva, Switzerland; ^8^Department of Medical, Surgical & Health Sciences, University of Trieste, Trieste, Italy; ^9^University Health Authority Giuliano-Isontina (ASUGI), Public Health Department, Trieste, Italy

**Keywords:** COVID-19, health protection, lock down, risk reduction, risk perception, attitude, adherence, social restrictions

## Abstract

**Background:** During the first wave of the COVID-19 pandemic (April to May 2020), 6,169 Polish and 939 Italian residents were surveyed with an online questionnaire investigating socio-demographic information and personality traits (first section) as well as attitudes, position, and efficacy perceptions on the impact of lockdown (second section) and various health protection measures enforced (third section).

**Methods:** The “health protection attitude score” (HPAS), an endpoint obtained by pooling up the answers to questions of the third section of the survey tool, was investigated by multiple linear regression models, reporting regression coefficients (RC) with 95% confidence intervals (95% CI).

**Results:** Concerns for business and health due to COVID-19 were associated with a positive attitude toward risk reduction rules. By contrast, male sex, concerns about the reliability of information available online on COVID-19 and its prevention, along with the feeling of not being enough informed on the transmissibility/prevention of SARS-CoV-2 were associated with a negative attitude toward risk mitigation measures.

**Discussion:** A recent literature review identified two social patterns with different features in relation to their attitude toward health protection rules against the spread of COVID-19. Factors positively associated with adherence to public health guidelines were perceived threat of COVID-19, trust in government, female sex, and increasing age. Factors associated with decreased compliance were instead underestimation of the COVID-19 risk, limited knowledge of the pandemic, belief in conspiracy theories, and political conservativism. Very few studies have tested interventions to change attitudes or behaviors.

**Conclusion:** To improve attitude and compliance toward risk reduction norms, a key intervention is fostering education and knowledge on COVID-19 health risk and prevention among the general population. However, information on COVID-19 epidemiology might be user-generated and contaminated by social media, which contributed to creating an infodemic around the disease. To prevent the negative impact of social media and to increase adherence to health protection, stronger content control by providers of social platforms is recommended.

## Introduction

On 11st March 2020 the World Health Organization (WHO) declared the COVID-19 pandemic, officially starting the so-called phase 1 (World Health Organization, 2021). Before then, on February 21, 2020 the first COVID-19 clusters in Northern Italy burst out, affecting 11 municipalities in the regions of Lombardy and Veneto (Reuters, 2021). Tougher social restrictions on the general population were progressively imposed in Italy until 9 March 2020, when a national lock down was declared. Several European countries enforced more or less similar social restrictions, with the aim of curbing the spread of SARS-CoV-2. Supplementary Table 1 “*Urgent nationwide measures to prevent and control the COVID-19 emergency*” lists the main regulations introduced in Italy (Italian Government, 2021). Similar coercive health protection measures were introduced also in Poland (Pinkas et al., 2020). These social limitations began to be lifted on 16th May 2020, marking the beginning of phase 2 of the pandemic.

Whilst isolation and quarantine are long-standing health protection measures against the spread of dangerous communicable diseases, a country lock-down entails a number of extraordinary interventions limiting the social freedom of the general population to protect the entire community against the risk of contagion (Official Gazzette of the Italian Republic, 2021). The only movements permitted during a country lock-down are those justified by work, health and primary needs (e.g., purchasing food) and return home. Moreover, a curfew is imposed from 6 pm on, social events (team sports, gyms, religious events, funerals, cinemas, museums, etc.) are suspended and smart working is encouraged. Activities of schools, including nurseries and universities are also suspended. Bars and restaurants, initially permitted to open between 6 am and 6 pm, were eventually allowed to continue their business only as take away or domiciliary delivery (Official Gazzette of the Italian Republic, 2021).

Social restrictions are somehow in contrast with the ethical and juridical principles of European countries, which are centered around liberty and autonomy of the individual. Nobody can be obliged to follow a health treatment unless imposed by law, in full respect of human rights (Benelhocine, 2021). Nonetheless, health protection measures against COVID-19 should take into account the principle of solidarity and the good of the entire community. The solidarity principle implies that the actions of the individual should be directed at protecting not only his/her own health, but also the general population. Therefore, the principle of solidarity recalls the principle of individual responsibility to adopt social behaviors protecting other people from SARS-CoV-2 contagion (Davies and Savulescu, 2019).

A survey on Albanian residents and expats reported a high satisfactory rate (88.0%) on infection prevention and control measures against COVID-19 (Kamberi, 2020). Likewise, an Indian survey from Kerala region revealed that 95% respondents were respectful of social restrictions imposed by the local government to tackle the current pandemic (Saji, 2020). By contrast, United Kingdom residents were less satisfied with risk reduction measures implemented against COVID-19, especially with bans affecting business activities, with 31.1% interviewees convinced that quarantine could cause health problems, independently from the country of residence (Saji, 2020).

Concerns for work and business are not the only factors influencing the perceptions of health protection measures against the spread of COVID-19. Misinformation, fake news and conspiracy theories inundating social media since the beginning of the COVID-19 pandemic have generated a worrisome “*infodemic*,” undermining the credibility of health institutions, at least in some sectors of the general population (The Lancet Infectious Disease, 2020).

Furthermore, attitude and compliance with health protection measures against the spread of COVID-19 could vary between and within countries.

In view of the above we investigated the attitude and perception of health protection measures against COVID-19 during the first pandemic wave in Italy and Poland, to inform public health policy makers on the respective determinants. Despite Italy being more heavily hit than Poland during the first COVID-19 wave, social restrictions in the latter two countries were in fact similar (Dong et al., 2020; Roser et al., 2020; Grabowski et al., 2021).

## Materials and Methods

During the first wave of COVID-19 pandemic (April – May 2020) Polish and Italian (Sardinian) residents were approached and surveyed by national/local media, regional websites, social media and university newsletters using an online questionnaire. A convenience snow-balling sampling was employed to approach as many respondents as possible from both countries. The survey instrument was posted online, participation to the survey was voluntary and all respondents returning a filled-up questionnaire were included in the study. A cross-sectional study design was adopted. Polish and Italian version of the survey tool were distributed.

The first section of the questionnaire collected socio-demographic information (Table 1). The second part investigated various perceptions/attitudes/positions (Table 2), using a Likert-type scale based upon four possible options (Grabowski et al., 2021). The last section included 7 questions (Table 3) on the attitude/perception of health protection rules, using pre-classified responses based on a Likert-type scale. The Cronbach alpha test for the seven outcome items displayed in Table 3 was calculated with Stata 14.2 and equaled 0.8141 for the overall sample, 0.8019 for the Italian version and 0.8164 for the Polish version.

**TABLE 1:** Distributions of socio-demographic information collected by the survey.

Variables	Categories	Total *N* (%) (*N* = 7,089)	Poland *N* (%) (*N* = 6,169)	Italy *N* (%) (*N* = 920)	*X*^2^ *p*-value
Sex	Female	5,285 (74.3)	4,698 (76.2)	587 (62.2)	<0.001
	Male	1,749 (24.6)	1,402 (22.7)	347 (36.8)	
	NOS	79 (1.1)	69 (1.1)	10 (1.1)	
Age (years)	18–24	2,036 (28.6)	1,945 (31.5)	91 (9.5)	<0.001
	25–34	2,277 (32.0)	2,034 (33.0)	243(25.9)	
	35–44	1,473 (20.7)	1,270 (20.6)	203 (21.7)	
	45–54	736 (10.4)	552 (9.0)	184 (19.4)	
	55–64	439 (6.2)	259 (4.2)	180 (19.3)	
	65+	147 (2.1)	109 (1.8)	38 (4.2)	
Place of residence	Village/town	909 (12.8)	692 (11.2)	217 (23.1)	<0.001
	Centre < 50,000 inhabitants	88 (12.4)	730 (11.8)	150 (16.0)	
	Centre 50,000–150,000 inhabitants	989 (13.9)	604 (9.8)	305 (41.0)	
	Centre 150,000–500,000 inhabitants	1,990 (28.0)	1,894 (30.7)	96 (10.2)	
	Centre > 500,000 inhabitants	2,341 (32.9)	2,249 (36.5)	92 (9.8)	
Number of household members	0	664 (9.3)	664 (9.3)	0	<0.001
	1	2,130 (30.0)	1,998 (32.4)	130 (14.2)	
	2	1,732 (24.4)	1,473 (23.9)	256 (27.6)	
	3	1,648 (23.2)	1,409 (22.8)	239 (25.5)	
	4	866 (12.2)	625 (10.1)	241 (25.5)	
	5+	67 (0.9)	0	67 (7.1)	
Marital status	Single	3,303 (46.4)	2,690 (43.6)	599 (65.0)	<0.001
	Married/relationship	3,215 (45.2)	3,166 (51.3)	49 (5.2)	
	Divorced/separated	270 (3.8)	255 (4.1)	15 (1.6)	
	Widow	324 (4.6)	58 (0.9)	256 (20.2)	
Building of residence	House	1,580 (22.2)	1,299 (21.1)	268 (29.2)	<0.001
	Terraced house	393 (5.5)	393 (6.4)	0	
	Condo	911 (12.8)	776 (12.6)	134 (14.6)	
	Apartment	4,101 (57.7)	3,589 (58.2)	504 (54.9)	
	Dorm/other	125 (1.8)	112 (1.8)	12 (1.3)	
Educational level	Junior 2 dairy school	1,868 (26.3)	1,834 (29.7)	31 (3.4)	<0.001
	Secondary school	366 (5.2)	76 (1.2)	283 (30.8)	
	Higher education	4,793 (67.4)	4,194 (68.0)	586 (63.7)	
	Professional qualification	86 (1.2)	65 (1.1)	20 (2.2)	
Occupational status	Employed (with contract)	3,474 (48.8)	3,002 (48.7)	463 (50.3)	<0.001
	Entrepreneur	596 (8.4)	481 (7.8)	110 (12.0)	
	Free lance	386 (5.4)	375 (6.1)	11 (1.2)	
	Worker without contract	16 (0.2)	0	16 (1.7)	
	Student	1,917 (27.0)	1,777 (28.0)	133 (14.5)	
	Unemployed	305 (4.3)	242 (28.8)	63 (6.7)	
	Pensioner	202 (2.8)	152 (2.5)	49 (5.3)	
	Other	217 (3.1)	140 (2.3)	75 (8.2)	
Work conditions	Business suspended	1,082 (15.2)	931 (15.1)	151 (16.1)	0.049
	Smart working	3,005 (42.3)	2,581 (41.8)	424 (45.1)	
	Business as usual	1,076 (15.1)	933 (15.1)	143 (152)	
	Not applicable	1,947 (27.4)	1,724 (28.0)	223 (23.7)	
Current financial condition	Good	1,660 (23.3)	1,543 (25.0)	116 (12.6)	<0.001
	Fairly good	4,188 (58.9)	3,589 (58.2)	585 (63.7)	
	Struggling to cope	879 (12.4)	716 (11.6)	157 (17.1)	
	Cannot cope	153 (2.2)	134 (2.2)	18 (2.0)	
	Not answer	231 (3.3)	107 (3.0)	43 (4.7)	
Self-perceived health status	Very fragile	24 (0.3)	17 (0.3)	6 (0.7)	<0.001
	Fragile	121 (1.7)	87 (1.4)	33 (3.6)	
	Average	879 (12.4)	719 (11.7)	155 (16.9)	
	Good	3,382 (47.6)	2,911 (47.2)	461 (50.2)	
	Very good	2,706 (38.1)	2,435 (39.5)	264 (38.7)	
Did you get diagnosed with COVID-19?	No	7,098 (99.8)	6,191 (99.6)	913 (99.2)	<0.001
	Yes	15 (0.2)	8 (0.1)	7 (0.8)	
Do you have respiratory symptoms now?	No	6,685 (94.0)	5,750 (93.2)	911 (99.0)	<0.001
	Yes	428 (6.0)	419 (6.8)	9 (1.0)	
Are you affected by chronic conditions?	No	4,784 (67.3)	4,023 (65.2)	742 (80.7)	<0.001
	Yes	2,329 (32.7)	2,146 (34.8)	178 (19.4)	
Some of your household members currently have respiratory symptoms	No	6,675 (93.9)	5,744 (93.1)	907 (98.7)	<0.001
	Yes	437 (6.1)	425 (6.9)	12 (1.3)	
Some of your household members has been diagnosed with COVID-19	No	6,980 (98.1)	6.079 (98.5)	877 (95.3)	<0.001
	Yes	133 (1.9)	90 (1.5)	43 (4.7)	
How would you define your character?	Not sociable	201 (2.8)	188 (3.1)	13 (1.4)	<0.001
	Partly unsociable	837 (11.8)	794 (12.9)	42 (4.6)	
	Neutral	2,030 (28.5)	1,794 (29.1)	229 (24.9)	
	Partly sociable	2,444 (34.4)	343 (37.3)	2,092 (33.9)	
	Very sociable	1,601 (22.5)	1,301 (21.1)	293 (31.9)	
How would you define your character?	Nervous	242 (3.4)	211 (3.4)	30 (3.3)	<0.001
	Partly nervous	1,061 (15.2)	989 (16.0)	91 (9.9)	
	Neutral	2,280 (32.1)	1,919 (31.1)	349 (37.9)	
	Partly calm	2,450 (34.4)	2,146 (35.8)	296 (32.2)	
	Calm	1,060 (14.9)	904 (14.7)	154 (16.7)	
How would you define your character?	Reserved	362 (5.1)	285 (4.6)	74 (8.1)	<0.001
	Partly reserved	1,408 (19.8)	1,180 (19.1)	220 (23.9)	
	Neutral	1,948 (27.4)	1,660 (26.9)	280 (30.7)	
	Partly extroverted	2,064 (29.0)	1,870 (30.3)	190 (20.7)	
	Extroverted	1,330 (18.7)	1,174 (19.0)	155 (16.9)	
How would you define your character?	Pessimist	309 (4.3)	271 (4.4)	37 (4.0)	0.018
	Partly pessimist	1,046 (14.7)	115 (12.5)	927 (15.0)	
	Neutral	2,122 (29.8)	1,856 (30.1)	263 (28.6)	
	Partly optimist	2,387 (33.6)	2,064 (33.5)	312 (21.0)	
	Optimist	1,249 (17.6)	1,051 (17.0)	193 (21.0)	

*Number (N) and column percentage (%). NOS, non-otherwise specified.*

**TABLE 2:** Distributions of attitudes/perceptions on health protection measures against the spread of COVID-19 during the first epidemic wave by country.

Questions	Categories	Answers	Total *N* (%)	Poland *N* (%)	Italy *N* (%)	*X*^2^ *p*-value
(1) Which of the following points worries you more in the near future?	My health status	No	788 (11.1)	707 (11.5)	79 (8.6)	<0.001
		Partly disagree	2,425 (34.1)	2,169 (35.2)	250 (273)	
		Partly agree	1,971 (27.7)	1,730 (28.0)	236 (25.8)	
		Yes	1,925 (27.1)	1,563 (25.3)	351 (38.3)	
	The health of people I care of	No	285 (4.0)	230 (3.7)	53 (5.8)	0.021
		Partly disagree	1,452 (20.4)	1,264 (20.5)	184 (20.0)	
		Partly agree	1,927 (27.1)	1,683 (27.3)	238 (25.9)	
		Yes	3,448 (48.5)	2,992 (48.5)	444 (48.3)	
	The economic stability of my family	No	1,095 (15.4)	982 (15.9)	110 (12.1)	<0.001
		Partly disagree	1,963 (27.6)	1,785 (28.9)	176 (19.3)	
		Partly agree	1,555 (21.9)	1,329 (21.5)	220 (24.1)	
		Yes	2,492 (35.1)	2,073 (33.6)	407 (44.6)	
	My Job	No	119 (1.7)	982 (15.9)	14 (1.5)	0.155
		Partly disagree	550 (7.7)	1,785 (28.9)	64 (7.0)	
		Partly agree	1,879 (26.4)	1,329 (21.5)	222 (24.2)	
		Yes	4,653 (64.2)	2,073 (33.6)	618 (67.3)	
	The economic stability of my country	No	84 (1.2)	73 (1.2)	9 (1.0)	<0.001
		Partly disagree	330 (4.6)	291 (4.7)	38 (4.1)	
		Partly agree	2,066 (29.1)	1,851 (30.0)	208 (22.7)	
		Yes	4,631 (65.1)	3,954 (64.1)	663 (72.2)	
	The stability of the global economic	No	123 (1.7)	107 (1.7)	14 (1.5)	0.067
		Partly disagree	668 (9.4)	575 (9.3)	88 (9.6)	
		Partly agree	2,603 (36.6)	2,295 (37.2)	300 (32.7)	
		Yes	3,716 (52.3)	3,192 (51.7)	515 (56.2)	
(2) Benefits of modern technologies on everyday life in home self-isolation	Possibility of working from home	Totally agree	3,760 (52.9)	2,983 (48.4)	759 (83.0)	<0.001
		Mostly agree	856 (12.0)	756 (12.3)	97 (10.6)	
		Rather disagree	260 (3.7)	223 (3.6)	36 (3.9)	
		Totally disagree	413 (5.8)	404 (6.6)	8 (0.9)	
		NA	1,818 (25.6)	1,803 (29.2)	14 (1.5)	
	Possibility of e-learning for mandatory training	Totally agree	2,833 (39.9)	2,126 (34.5)	689 (75.6)	<0.001
		Mostly agree	1,238 (17.4)	1,097 (17.8)	140 (15.4)	
		Rather disagree	408 (5.7)	349 (5.7)	56 (6.2)	
		Totally disagree	314 (4.4)	300 (4.9)	13 (1.4)	
		NA	2,311 (32.5)	2,27 (37.2)	13 (1.4)	
	Possibility of remote contact with friends/relatives	Totally agree	4,708 (66.3)	3,973 (64.4)	717 (78.7)	<0.001
		Mostly agree	1,719 (24.2)	1,586 (25.7)	127 (13.9)	
		Rather disagree	381 (5.4)	339 (5.5)	42 (4.6)	
		Totally disagree	216 (3.0)	197 (3.2)	19 (2.1)	
		NA	80 (1.1)	74 (1.2)	6 (0.7)	
	Leisure activities online (movies, theatre, TV, music concerts, etc.)	Totally agree	4,730 (66.6)	3,952 (64.1)	759 (83.3)	<0.001
		Mostly agree	1,685 (23.7)	1,567 (25.4)	113 (12.4)	
		Rather disagree	433 (6.1)	411 (2.8)	22 (2.4)	
		Totally disagree	185 (2.6)	171 (2.8)	14 (1.5)	
		NA	71 (1.0)	68 (1.1)	3 (0.3)	
	Retrieval of online information on COVID-19 and its prevention	Totally agree	4,265 (60.1)	3,588 (58.2)	677 (72.6)	<0.001
		Mostly agree	1,990 (28.0)	1,798 (29.2)	192 (20.6)	
		Rather disagree	495 (7.0)	456 (7.4)	39 (4.2)	
		Totally disagree	249 (3.5)	229 (3.7)	20 (2.1)	
		NA	103 (1.5)	98 (1.6)	5 (0.5)	
(3) To which extent would you agree with the following points?	Health support for quarantined individuals is adequate	Totally agree	239 (3.4)	170 (2.8)	65 (7.1)	<0.001
		Fairly agree	1,071 (15.1)	768 (12.5)	296 (32.0)	
		Rather disagree	1,930 (27.1)	1,675 (27.2)	247 (26.9)	
		Totally disagree	1611 (22.7)	1,482 (24.0)	128 (13.9)	
		NA	2,260 (31.8)	2,074 (33.6)	182 (19.8)	
	Access to food, essential and personal hygiene goods is adequate for quarantined individuals	Totally agree	436 (6.1)	233 (3.8)	196 (21.4)	<0.001
		Fairly agree	1,693 (23.8)	1,368 (22.2)	318 (34.6)	
		Rather disagree	1,274 (17.9)	1,137 (18.4)	131 (14.3)	
		Totally disagree	826 (11.6)	775 (12.6)	50 (5.5)	
		NA	2,882 (40.5)	2,656 (43.1)	223 (24.3)	
	The financial support for quarantined individuals is adequate	Totally agree	146 (2.1)	107 (1.7)	37 (4.1)	<0.001
		Fairly agree	574 (8.1)	412 (6.7)	158 (17.3)	
		Rather disagree	1,569 (22.1)	1,302 (21.1)	261 (28.6)	
		Totally disagree	2,129 (30.0)	1,879 (30.5)	243 (26.6)	
		NA	2,688 (37.8)	2,469 (40.0)	214 (23.4)	
	I have access to reliable epidemiological information on the situation of my country	Totally agree	1,313 (18.5)	1,040 (16.9)	266 (29.0)	<0.001
		Fairly agree	2,350 (33.1)	1,987 (32.2)	355 (38.7)	
		Rather disagree	1,423 (20.0)	1,252 (20.3)	168 (18.3)	
		Totally disagree	1,224 (17.2)	1,130 (18.3)	90 (9.8)	
		NA	801 (11.3)	760 (12.3)	39 (4.3)	
	I feel enough informed on the transmissibility of SARS-CoV-2	Totally agree	1,829 (25.7)	1,432 (23.2)	388 (42.2)	<0.001
		Fairly agree	2,799 (39.4)	2,429 (39.4)	361 (39.8)	
		Rather disagree	1,132 (15.9)	1,027 (16.7)	104 (11.3)	
		Totally disagree	942 (13.3)	894 (14.3)	44 (4.8)	
		NA	410 (5.8)	387 (6.3)	22 (2.4)	
	Quarantined or isolated individuals in financial difficulties should receive economic support	Totally agree	4,286 (60.3)	3,545 (57.5)	724 (79.0)	<0.001
		Fairly agree	2,067 (29.1)	1,911 (31.0)	150 (16.4)	
		Rather disagree	257 (3.6)	231 (3.7)	25 (2.7)	
		Totally disagree	160 (2.3)	152 (2.5)	8 (0.9)	
		NA	340 (4.8)	330 (5.4)	10 (1.1)	

*Number (N) and column percentage (%).*

**TABLE 3:** Attitudes/perceptions on health protection measures against the spread of COVID-19 by level of agreement and country of residence (Number, N and percentage, %); and Health Protection Attitude Score (Mean, M ± standard deviation, SD) by country.

Items	Level of agreement	Total	Poland		Italy	
			*N* (%)	*M* ± *SD*	*N* (%)	*M* ± *SD*
(1) The NHS is entitled to enforce social restrictions of COVID-19 contacts for good reasons	Agree	3,836 (53.9)	3,177 (51.5)	2.39 ± 0.74	645 (70.1)	2.64 ± 0.62
	Partly agree	2,724 (38.3)	2,481 (40.2)		233 (25.3)	
	Partly disagree	335 (4.7)	309 (5.0)		26 (2.8)	
	Disagree	218 (3.1)	202 (3.3)		16 (1.7)	
(2) Social distancing and home isolation are effective measures to control the spread of COVID-19	Agree	3,830 (53.9)	3,177 (51.5)	2.37 ± 0.77	639 (69.6)	2.61 ± 0.67
	Partly agree	2,564 (36.9)	2,333 (37.8)		222 (24.2)	
	Partly disagree	465 (6.5)	429 (7.0)		36 (3.9)	
	Disagree	252 (3.5)	230 (3.7)		21 (2.3)	
(3) A quarantined individual should always obey the rules	Agree	5,616 (79.0)	4951 (80.3)	2.74 ± 2.63	649 (70.6)	2.63 ± 0.66
	Partly agree	1,173 (16.5)	949 (15.4)		217 (23.6)	
	Partly disagree	168 (2.4)	134 (2.2)		33 (3.6)	
	Disagree	155 (2.2)	135 (2.2)		20 (2.2)	
(4) Quarantine protects household members from COVID-19 contagion	Agree	3.502 (49.3)	2,886 (46.8)	2.17 ± 0.93	603 (65.6)	2.52 ± 0.76
	Partly agree	2,128 (29.9)	1,901 (30.8)		216 (23.5)	
	Partly disagree	1,029 (14.5)	957 (15.5)		72 (7.8)	
	Disagree	452 (6.4)	425 (6.9)		27 (2.9)	
(5) Quarantine protects surrounding individuals from COVID-19 contagion	Agree	4,308 (60.6)	3,685 (59.7)	2.46 ± 0.77	609 (66.5)	2.54 ± 0.74
	Partly agree	2,089 (29.4)	1,864 (30.2)		216 (23.6)	
	Partly disagree	47 (6.7)	404 (6.6)		69 (7.5)	
	Disagree	239 (3.4)	216 (3.5)		22 (2.4)	
(6) Breach of quarantine measures should be prosecuted and punished	Agree	4,270 (60.0)	3,705 (60.1)	2.44 ± 0.81	552 (60.1)	2.44 ± 0.80
	Partly agree	2,084 (28.2)	1,731 (28.1)		264 (28.7)	
	Partly disagree	521 (7.3)	457 (7.4)		62 (6.8)	
	Disagree	317 (4.5)	276 (4.5)		41 (4.5)	
(7) The NHS should be allowed to enforce quarantine measures even without consent	Agree	3,671 (51.6)	3,175 (51.5)	2.28 ± 0.88	485 (52.3)	2.29 ± 0.90
	Partly agree	2,208 (31.1)	1,922 (31.2)		277 (30.1)	
	Partly disagree	818 (11.5)	721 (11.7)		95 (10.3)	
	Disagree	414 (5.8)	351 (5.7)		62 (6.8)	

National laws and ethical guidelines for studies involving human subjects were observed. Ethics approval was obtained from the Independent Bioethical Commission for Issues of Scientific Research at the University of Gdańsk (Resolution Number NKBBN/144/2021). In particular, interviewees provided an electronic informed consent before completing the questionnaire (Supplementary Files 1, 2). The study was conducted in accordance with the ethical principles of the Declaration of Helsinki.

### Statistical Analysis

#### Variables

All answers to questions displayed in Table 1 were considered categorical variables. Pre-classified answers to questions listed in Table 2 were Likert-type items which were coded as follows: “No = 0, Partly disagree = 1, Partly agree = 2, Yes = 3” (question 1); and “Totally agree = 0, Fairly agree = 1, Rather disagree = 2, Totally disagree = 3 and NA = 4” (questions 2 and 3). The latter were considered as factor variables (*i.varname, according to STATA syntax*) in the statistical analysis.

The answers to questions related to attitude and perception of health protection rules (Table 3) were assigned the following values: “Agree = 3; Partly agree = 2; Partly disagree = 1; and Disagree = 0.” The scores developed for each of the above 7 questions (ranging from 0 to 3) were summed up to obtain a pooled linear indicator, which we defined “*health protection attitude score*” (HPAS). Attitude/efficacy perception toward health protection rules increased with HPAS, which could reach a maximum value of 21 (=7 × 3).

#### Analysis

Numbers and percentage were calculated for each available term.

A multiple linear regression was fitted, where the outcome was HPAS and predictors were the above variables included in Tables 1, 2. The independent variables included in the multiple regression model were selected by using an automated stepwise procedure. Results were expressed as regression coefficients (RC) with 95% confidence interval (95% CI). We used the procedure of Benjamini–Hochberg (BH) to control for the false discovery rate when conducting multiple comparisons (Benjamini and Hochberg, 1995). Finally, using the values returned by the computer program, the predicted values of HPAS were calculated (Y = *a* + *b*_1_*x*_1_ + *b*_2_*x*_2_) for different values of predictors, using the Stata command “predict,” after fitting the final multivariable linear regression model, adding also the constant term to the predicted value estimated.

Stata 14.2 (Stata corporation, College Station, TX, United States) was used for the analysis.

## Results

A total number of 7,108 questionnaires were returned: 6,169 from Poland (77% females vs. 23% males) and 939 from Sardinia (62,8% females vs. 37,1% males).

Table 1 shows the distribution of socio-demographic variables. As can be seen, interviewees were predominantly Polish residents (*N* = 6,169; 87.0%) than Italians (*N* = 920) and 74.3% were females. The age groups more represented were 25–34 years old (32.0%), followed by 18–24 years old (28.6%) and 35–44 years old (20.7%). Most respondents were living in centers with >500,000 (32.9%) or 150,000–500,000 (28.0%) inhabitants and were either single (46.4%) or married/in a relationship (45.2%) and were living with 1 (30.0%) or 2 (24.4%) household members. The vast majority of interviewees were living in apartments (57.7%) followed by condos (12.8%) and were students (27.0%), with higher education (67.4%) and were doing smart-working (42.3%). Most interviewees were in fairly good (58.9%) or good (23.3%) financial conditions and perceived their health conditions as good (47.6%) or very good (38.1%). About 32.7% of individuals were affected by co-morbidities, with 0.2% of those diagnosed with COVID-19 and 6% with respiratory symptoms at the time of the interview. Respondents predominantly had a “*partly social*” (34.4%), “*partly calm*” (34.4%), “*partly extroverted*” (29.0%) or “*partly optimist”* (33.6%) character trait.

Table 2 shows the distribution of variables expressing the perceptions as well as attitudes on health protection measures during the first pandemic wave of COVID-19. There were three main questions, each including several sub-questions (categories) with the corresponding pre-classified options (answers). The main results are the following:

Higher concerns were reported for “*the economic stability of my country*” (Yes = 65.1%), “*my job*” (Yes = 64.2%), “*stability of the global economic*” (Yes = 52.3), and “*health of people I care of*” (Yes = 48.5%).

Regarding benefits of modern technologies, “*Totally agree*” was expressed by 66.6% interviewees (on leisure activities online), 66.3% (on remote contact with friends/relatives), 60.1% (on retrieval of information online) and 52.9% (working from home); the corresponding percentage was, however, 39.9% for the “*Possibility of e-learning for mandatory training.*”

For three questions asking “*is … adequate for quarantined individuals*?”, the more common answer was “NA,” with a percentage equal to 40.5% (regarding access to food and essential goods), 37.8% (on financial support), and 31.8% (on health support). Obviously, for 60.3% of interviewees, quarantined individuals “should receive economic support.”

Regarding access to reliable epidemiological information, there was not a sharp contrast in beliefs but a set of opinions. The sum of “*Totally agree*” and “*Fairly agree*” is always higher than 50%, but “*Totally disagree*” is 17.2% for one question and 13.3% for the other.

Table 3 shows the personal positions on norms to control the spread of COVID-19 by country and level of agreement (number and percentage of respondents); and the value (mean ± standard deviation) of HPAS by country. The main interest of this table is the chance of estimating the weight of different groups. “*Agree*” with the 7 questions was expressed by 58% (min-max: 49%; 79%), while “*Disagree*” was reported by 4% (min–max: 2%; 6%) of interviewees.

As can be seen there was a highly significant cross-country variability in the distribution of all factors displayed in Tables 1–3, with the exception of occupational status of interviewees (*p* = 0.049) and “*concerns for my job*” (*p* = 0.155) and “*stability of the global economy*” (*p* = 0.067) in the near future (Table 3).

Table 4 displays the results of the multiple linear regression model, fitted onto 6,992 complete observations, on the attitude/perception toward health protection norms. It can be seen that, apart from sex, socio-demographic factors (shown in Table 1) were not significantly associated with HPAS. The three questions listed in Table 2 were the main drivers. Each of the latter had Likert-type items as pre-classified answers. If one or more Likert items showed non-significant RCs, the whole cluster was dropped from Table 4. Otherwise, if all RCs were significant according to BH procedure, their signs were consistent (always positive or negative) and their values displayed a monotonic trend. The sign of RC specifies the direction of association (positive sign indicating higher attitude/perception of health protection rules, and vice versa); the value of RC and the statistical significance expresses the strength of association; the monotonic trend of predicted values of HPAS displays an exposure-effect relationship. Furthermore, since they were adjusted for all other variables, RCs were devoid of confounding effect, fulfilling Hill’s criteria of causality.

**TABLE 4:** Multiple linear regression model.

Questions	Options	Likert items	RC (95% CI)	BH *p*-value	($\hat{\text{y}}$).
(1) Which of the following points worries you more for the near future?	My job	No	Reference		
		Partly disagree	0.44 (0.19; 0.69)	2.63 E-08	13
		Partly agree	2.42 (1.85; 2.96)	3.32 E-15	18
		Yes	2.94 (2.38; 3.51)	3.95 E-22	21
	My health status	No	Reference		
		Partly disagree	0.44 (0.19; 0.69)	0.0026	13
		Partly agree	0.60 (0.33; 0.87)	0.0001	14
		Yes	0.88 (0.60; 1.17)	2.37 E-08	15
(2) Benefits of modern technologies on everyday life in home self-isolation	Retrieval of online information on COVID-19 and its prevention	Totally agree	Reference		
		Mostly agree	−0.41 (−0.58; −0.24)	1.90 E-05	12
		Rather disagree	−1.12 (−1.41; −0.84)	2.34 E-13	10
		Totally disagree	−1.37 (−1.76; −0.97)	2.37 E-10	9
		NA	−1.02 (−1.60; −0.43)	0.0032	
(3) To which extent would you agree with the following points?	I feel enough informed on the transmissibility of SARS-CoV-2	Totally agree	Reference		
		Fairly agree	−0.24 (−0.44; −0.05)	0.0428	12
		Rather disagree	−0.52 (−0.77; −0.28)	0.0002	12
		Totally disagree	−0.72 (−1.00; −0.43)	8.09 E-06	11
		NA	−0.51 (−0.88; −0.15)	0.0249	
Sex	Female		Reference		
	Male		−0.28 (−0.44; −0.12)	0.0033	

*Outcome: Health Protection Attitude Score (HPAS). Regression coefficients (RC) with 95% confidence intervals (95%CI) and Benjamini-Hochberg (BH) p-values. Predicted value of HPAS [($\hat{y}$].*

Therefore, as can be seen in Table 4, concerns for business and health due to COVID-19 were associated with a positive attitude/perception toward health protection rules. By contrast, male subjects, concerns about reliability of information available online on COVID-19 and its prevention as well as feeling of not being enough informed on the transmissibility of SARS-CoV-2 were associated with a negative attitude for risk reduction measures.

## Discussion

### Key Findings

A relevant finding of the present study was that, albeit significant cross-country variability between Poland and Italy in the distribution of information collected, apart from sex, socio-demographic factors (displayed in Table 1) were not significantly associated with the endpoint HPAS, whereas perceptions and positions (listed in Table 2) were its main drivers. Despite future concerns for their work and health, individuals positively perceived the social restrictions imposed in Italy and Poland to control the spread of COVID-19. However, a negative attitude toward these measures was expressed by interviewees undervaluing the reliability of the available information on the transmissibility of SARS-CoV-2 and the benefits of modern technology to mitigate the inconveniences caused by home isolation.

### Study Limitations

This survey, relied on data collected via internet, cannot be considered representative of the respective general populations of Italy and Poland. In fact, our convenience sample was mainly composed of females, Polish respondents, <44 years of age. Thus, a selection bias cannot be excluded. However, the relatively large sample size, the wide range of explanatory variables and the employment of the BH criterium for statistical significance to filter possible false associations empowered this study to draw some relevant conclusions from this study.

### Generalizability

Two groups of interviewees could be distinguished from the present investigation, based upon the final multiple regression model: one that, due to the threat posed by COVID-19 on health and business activities, and despite present and future hardships, accept social restrictions imposed by the government with the goal of decreasing the circulation of SARS-CoV-2; a second group not accepting limitations of liberty under the justification that the current epidemiologic information on COVID-19 is not reliable (Table 4).

Likewise, in a review of recent studies, two groups of individual characteristics were identified in relation to their attitude/compliance with public health measures against the spread of COVID-19 (Vanhove et al., 2015; Moran et al., 2021):

• GROUP 1 (“Eudaimonic”): perceiving the threat of COVID-19, trusting governments, more likely to be female and older, consistently showing a positive attitude/adherence with public health guidelines. Factors positively related to adherence though less frequently mentioned were higher socio-economic status, accessing traditional media sources, trust in science or medicine, perceived effectiveness of risk reduction measures, ability to follow guidelines.
• GROUP 2 (“Hedonic”): characterized by decreased adherence to public health guidelines, political conservatism and belief in conspiracy theories. Those with limited knowledge of the pandemic, those who felt that COVID-19 posed a low risk, and those who were unconvinced of the efficacy of health protection measures to contrast the spread of SARS-CoV-2 were consistently more likely to exhibit poor adherence.

In a study carried out in representative samples across eight countries (*N* = 7,568), the additional mortality rate over a 3-month period (June–August 2020) following the enforcement of lock-down against the spread of COVID-19 was 10 times higher in countries with low adherence against risk mitigation measures (United States, Sweden, Poland, and Russia) than in those with high compliance (Germany, France, Spain, and United Kingdom) (Margraf et al., 2021). Cross-country discrepancies in the adherence with health protection measures correlated with subsequent increase of mortality (correlation coefficient = −0.91) (Margraf et al., 2021). Cross-country modeling of social restrictions showed that implementing non-pharmaceutical interventions (NPIs) determines an instantaneous reduction of the base reproduction number (*R*_*t*_)−an indicator of new infections of SARS-CoV-2 (Brauner et al., 2021). Brauner et al. (2021) gathered chronological data on the implementation of NPIs for 41 European and non-European countries between January and end of May 2020. The effectiveness of these NPIs was estimated by linking their enforcement dates with national case and death counts. Shutting down all educational institutions, limiting social gatherings to 10 people or less and suppressing face-to-face businesses considerably reduced the transmission of SARS-CoV-2. The additional effect of the stay-at-home policy on the transmissibility of SARS-CoV-2 was instead comparatively marginal (Brauner et al., 2021). Finally, mathematical modeling suggested that while delaying the imposition of social restrictions by 1 or 2 weeks had a negligible effect on the circulation of SARS-CoV-2, an earlier relaxation of these measures by 1 or 2 weeks translated into a marked increase of the respective infection rate (Gevertz et al., 2021).

There is a gap in the literature between the important scientific advances in COVID-19 knowledge and the strategies to promote adherence with health protection measures to contain this disease. In fact, according to the above-mentioned review (Moran et al., 2021), very few interventional studies or quasi-experimental studies have been published to date. Authors generally offered logical suggestions based on inferential findings from convenience sample surveys, rather than evidence from evaluations of interventions implemented to change attitudes and social behaviors (Moran et al., 2021).

Several people use social media platforms such as Facebook, Youtube, and Twitter as a source of information on COVID-19 (Allington et al., 2020; Brailovskaia et al., 2021; Moran et al., 2021). Differently from other sources (scientific journals, newspapers, television, reports, and official sites of governments and health authorities), the content available on social media is user-generated, hence each user can freely create, modify and share this content (Brailovskaia et al., 2021). As a consequence, social media often provide a huge amount of unfiltered (mis-) information and fake news (Allington et al., 2020; Cuello-Garcia et al., 2020; Pennycook et al., 2020; Brailovskaia et al., 2021). Previous research showed that the absorption of such information can have a major influence on the perceived burden of COVID-19, its related emotional overload, stress symptoms associated and poor adherence to urgent risk mitigation measures (Brailovskaia et al., 2021). To prevent the negative impact of social media use and to increase adherence to NPIs, a stronger content control on social platforms by the respective providers is urgently recommended. To further develop and disseminate solutions and resilience against mis- and disinformation, as requested in a “Joint Statement” by WHO, UN, UNICEF, UNDP, UNESCO, UNAIDS, ITU, UN Global Pulse and IFRC, all stakeholders – including media and social media, researchers, technologists capable to design and build effective strategies and tools to respond to the so called “infodemic,” civil society leaders and influencers – should cooperate with the UN system, with Member States and with each other (UNICEF, 2021).

## Conclusion

To improve attitude and compliance with risk reduction norms, a key intervention is fostering education and knowledge on the COVID-19 risk among the general population. However, a considerable proportion of information on COVID-19 epidemiology is user-generated and contaminated by social media, contributing to create an “infodemic” around this disease. It is therefore urgent to set up interventions to contain the mis- and disinformation disseminated online by users of social media. In addition, governmental communication should stress the responsibility of users for the contents they spread via social media and the need for the respective providers to verify all information posted online, before sharing it.

## Data Availability Statement

The raw data supporting the conclusions of this article will be made available by the authors, without undue reservation.

## Ethics Statement

The studies involving human participants were reviewed and approved by Independent Bioethical Commission for Issues of Scientific Research at the Medical University of Gdańsk (NKBBN/144/2021). The patients/participants provided their written informed consent to participate in this study.

## Author Contributions

LL, JG, JS, TM, and PW: study concept. LC and GM: study design, data analysis, and data interpretation. RM, LL, and JG: survey translation. RM, DP, SB, JG, JS, TM, and PW: data collection. LC, GM, LL, and DP: literature search. LC, GM, SB, and LL: manuscript drafting. All authors contributed to the article and approved the submitted version.

## Conflict of Interest

The authors declare that the research was conducted in the absence of any commercial or financial relationships that could be construed as a potential conflict of interest.

## Publisher’s Note

All claims expressed in this article are solely those of the authors and do not necessarily represent those of their affiliated organizations, or those of the publisher, the editors and the reviewers. Any product that may be evaluated in this article, or claim that may be made by its manufacturer, is not guaranteed or endorsed by the publisher.
