# Supplementary material for: Attitudes and Perceptions of Health Protection Measures Against the Spread of COVID-19 in Italy and Poland
Source: Front Psychol. 2021 Dec 24;12:805790. doi: 10.3389/fpsyg.2021.805790 (PMC8754188; doi:10.3389/fpsyg.2021.805790)
Supplement: Supplementary file 1 [file Table_1.DOCX]

**Supplementary Table 1 (S1). Public health meaasure enforced to mitigate the spread of SARS-CoV-2 in Italy (as of April 10, 2020)**

| **TYPE OF MEASURE** | **DETAILS** | **DATE ENACTED** | **ENFORCED** |
| --- | --- | --- | --- |
| State of emergency | - Deliberation of the state of emergency for six months | 31 January | 31 January |
| Ministry of Health Communique on quarantine | - Compulsory isolation measures (quarantine) for close contacts with a positive patient - Trustworthy isolation for those coming from risk areas over the last 14 days and mandatory self-notification to health authorities | 21 February | 21 February |
| Law Decree and Decree of the President of the Council of Ministries (DPCM)  (first regional lockdown measures) | Specific measures for certain municipalities located in Lombardy and Veneto (“red areas”):   - Ban on moving from and to the municipality - Ban on public and private events, including religious celebrations - Closing of every school and university, including ban on school trips, as well as museums and other cultural inistitutes, public offices, commercial activities (excluding essential services) - Mandatory use of individual protection devices when entering essential services - Suspension of public and private transportation systems - Implementation of smart working | 23 February | 23 February |
| DPCM  (full lockdown) | - Extension of the “red area” to the whole national territory (see above). Lockdown until 3 April | 9 March | 9 March |
| DPCM  (Extension of social restrictions) | - Closing of all commercial shop except for essential services | 11 March | 11 March |
| Ministry of Health  Further restrictions | - Closing of parks, limitation of physical activities within the immediate proximity of one’s home, closing of all activities selling food and beverage located in train and gas stations, ban on moving towards holiday houses. | 20 March | 21 March |
| DPCM  (Extension of lockdown and first relaxation of measures) | - Extension of the lockdown until 3 May - Opening of stationery stores, bookshops and shops for children and infants | 10 April | 14 April |
