# Supplementary material for: Attitudes and Perceptions of Health Protection Measures Against the Spread of COVID-19 in Italy and Poland
Source: Front Psychol. 2021 Dec 24;12:805790. doi: 10.3389/fpsyg.2021.805790 (PMC8754188; doi:10.3389/fpsyg.2021.805790)
Supplement: Supplementary file 2 [file Image_1.pdf]

MEDICAL
UNIVERSITY
OF GDAŃSK

www.mug.edu.pl
58 349 11 11
info@gumed.edu.pl
ul. M. Skłodowskiej-Curie 3a,
80-210 Gdańsk

Dear respondents,

With recent onset of pandemic, we are currently facing an unprecedented situation that affects our everyday life.

In this study we are asking you for your opinion on the requirements of social isolation during Co-VID-19 epidemic outbreak, as well as for the description on how isolation, quarantine and other disturbances influence your comfort.

Results will help to recognize how separation from other people affects health and mental state. We kindly request you to spend about 10 minutes answering the questions. Every single response is valuable to us. All data collected would be for use for scientific purposes only. Personal data will be confidential, coded for statistical analyses. Taking part in our query is voluntary. The only restriction is age above 18.

- Quarantine- obligatory separation of an individual suspected of being a host of an infectious disease
- Home isolation-restriction in contact or lack of direct social intercommunication (face-to-face contact)

How would you assess your health status?

- Very good
- Good
- Average
- Bad
- Very bad

Which of the selected conditions below have you experienced lately

- -I was irritated because something unexpected happened

  □ never, □ sometimes, □ frequently, □ very often

- -I feel that important matters in my life are becoming out of my control

  □ never, □ sometimes, □ frequently, □ very often

- -I feel agitation and anxiety

  □ never, □ sometimes, □ frequently, □ very often

- -I'm convinced that I can handle my personal matters

  □ never, □ sometimes, □ frequently, □ very often

- -Events are not going as I would like to

  □ never, □ sometimes, □ frequently, □ very often

- -I cannot take care of my responsibilities

  □ never, □ sometimes, □ frequently, □ very often

- -I can manage my annoyance

  □ never, □ sometimes, □ frequently, □ very often

- -I have a feeling that everything is going well

  □ never, □ sometimes, □ frequently, □ very often

- -I was angry because I had no influence on something that happened

  □ never, □ sometimes, □ frequently, □ very often

- -I feel like I cannot succeed in dealing with increasing difficulties

  □ never, □ sometimes, □ frequently, □ very often

How would you describe your character:

- Unsociable-sociable
- Anxious-calm
- reserved- outgoing-
- Pessimistic-optimistic

Which one of the sentences below describes best your beliefs:

- -Public healthcare institutions can issue restrictions in contacts in reasonable cases

  □ disagree , □ rather disagree , □ rather agree , □ agree

- -Home/social isolation is an effective way of limiting new cases of infectious diseases

  □ disagree , □ rather disagree , □ rather agree , □ agree

- -Every person should have the right to know why home isolation is introduced

  □ disagree , □ rather disagree , □ rather agree , □ agree

- -People under isolation should have right to psychological support

  □ disagree , □ rather disagree , □ rather agree , □ agree

- -A person put under quarantine should obey the rules despite other matters
  □ disagree , □ rather disagree , □ rather agree , □ agree

- -A person who subordinates quarantine effectively protects his own family from contagion
  □ disagree , □ rather disagree , □ rather agree , □ agree

- People who subordinate quarantine effectively protect immediate surroundings from contagion
  □ disagree , □ rather disagree , □ rather agree , □ agree

- -People, who disobey quarantine should be punished
  □ disagree , □ rather disagree , □ rather agree , □ agree

- -Public healthcare institutions should have the right to force someone under quarantine if a person does not consent voluntarily
  □ disagree , □ rather disagree , □ rather agree , □ agree

Have you got symptoms of respiratory tract infection at the moment?

□ yes □ no

Have you been diagnosed with Coronavirus disease?

□ yes □ no

Has somebody from your relatives got symptoms of respiratory tract infection at the moment?

□ yes □ no

MEDICAL
UNIVERSITY
OF GDAŃSK

www.mug.edu.pl
58 349 11 11
info@gumed.edu.pl
ul. M. Skłodowskiej-Curie 3a,
80-210 Gdańsk

Has somebody from your relatives been diagnosed with Coronavirus disease?

□ yes □ no

Are you diagnosed with chronic diseases?

□ yes □ no

If you might be put under quarantine which place would you prefer?

- my own flat or house
- in a place indicated by public services

If somebody from your relatives might be put under quarantine, which place would you prefer?

- my own flat or house
- in a place indicated by public services

Have you ever been under quarantine or are you now?

- I'm under quarantine, others from my household are not
- I'm under quarantine and my relatives from my household are under quarantine
- no

Which of the following statements do you agree with in regards to last month?

- I occasionally do some essential grocery shopping (1-2 times a week):
  □ definitely agree, □ mostly agree, □ rather disagree, □ definitely disagree

- I go out primarily to work:
  □ definitely agree, □ mostly agree, □ rather disagree, □ definitely disagree

MEDICAL UNIVERSITY OF GDAŃSK

www.mug.edu.pl
58 349 11 11
info@gumed.edu.pl
ul. M. Skłodowskiej-Curie 3a,
80-210 Gdańsk

- I go out for a walk in my neighborhood:

  □ definitely agree, □ mostly agree, □ rather disagree, □ definitely disagree

- I go out for a walk to public places (parks, beaches, boulevard etc.):

  □ definitely agree, □ mostly agree, □ rather disagree, □ definitely disagree

- Everyday I go out with my child/pet:

  □ definitely agree, □ mostly agree, □ rather disagree, □ definitely disagree

- I do sports outside (running, cycling etc):

  □ definitely agree, □ mostly agree, □ rather disagree, □ definitely disagree

- I regularly go to church:

  □ definitely agree, □ mostly agree, □ rather disagree, □ definitely disagree

- I regularly meet with friends:

  □ definitely agree, □ mostly agree, □ rather disagree, □ definitely disagree

- I only go out with most essential matters:

  □ definitely agree, □ mostly agree, □ rather disagree, □ definitely disagree

In what range do modern communication/information technologies have positive impact on your everyday life in the situation of home isolation.

- Possibility to work from home:

  □ definitely yes, □ mostly yes, □ rather no, □ definitely no, □ not applicable

- Possibility to realize obligatory education/learning from home:

  □ definitely yes, □ mostly yes, □ rather no, □ definitely no, □ not applicable

MEDICAL
UNIVERSITY
OF GDAŃSK

www.mug.edu.pl
58 349 11 11
info@gumed.edu.pl
ul. M. Skłodowskiej-Curie 3a,
80-210 Gdańsk

- Remote contact with relatives/friends:

  □ definitely yes, □ mostly yes, □ rather no, □ definitely no, □ not applicable

- Spare time activities (movies, tv series, online concerts etc):

  □ definitely yes, □ mostly yes, □ rather no, □ definitely no, □ not applicable

- Represent essential information source about spread of Coronavirus, preventive actions etc:

  □ definitely yes, □ mostly yes, □ rather no, □ definitely no, □ not applicable

Which of the following statements best represents your opinion?

- Health support for people under quarantine is on appropriate level:

  □ definitely agree, □ mostly agree, □ rather disagree, □ definitely disagree, □ difficult to say

- Life support (food, hygienic products) for people under quarantine is on appropriate level:

  □ definitely agree, □ mostly agree, □ rather disagree, □ definitely disagree, □ difficult to say

- Financial support for people under quarantine is on appropriate level:

  □ definitely agree, □ mostly agree, □ rather disagree, □ definitely disagree, □ difficult to say

- I have access to reliable information about epidemiological situation in country:

  □ definitely agree, □ mostly agree, □ rather disagree, □ definitely disagree, □ difficult to say

- I feel well informed about ways that virus spreads:
  □ definitely agree, □ mostly agree, □ rather disagree, □ definitely disagree, □ difficult to say

- People in tough financial situation, caused by home isolation, should receive financial support:
  □ definitely agree, □ mostly agree, □ rather disagree, □ definitely disagree, □ difficult to say

Which of the following aspects raises biggest concerns about the nearest future?

- Own health:
  □ definitely yes, □ mostly yes, □ rather no, □ definitely no

- Health of loved one:
  □ definitely yes, □ mostly yes, □ rather no, □ definitely no

- Your family financial stability:
  □ definitely yes, □ mostly yes, □ rather no, □ definitely no

- Own employment:
  □ definitely yes, □ mostly yes, □ rather no, □ definitely no

- Economic situation in Your country:
  □ definitely yes, □ mostly yes, □ rather no, □ definitely no

- Global economic situation:
  □ definitely yes, □ mostly yes, □ rather no, □ definitely no

MEDICAL
UNIVERSITY
OF GDAŃSK

www.mug.edu.pl
58 349 11 11
info@gumed.edu.pl
ul. M. Skłodowskiej-Curie 3a,
80-210 Gdańsk

Personal Information:

Age: ____________________

Sex:

- male,
- female,
- refuse to tell,
- other

Marital status:

- single,
- married/long term relationship,
- divorced,
- widow

Education:

- primary education,
- junior high school,
- vocational,
- secondary,
- higher

Employment status:

- student,
- employment contract,
- contract of mandate,
- work without a contract,
- own business,
- unemployed,
- pension,
- other

Do you currently work from home?

- Yes, I work from home,
- No, I still work from office,
- No, work was temporarily suspended,
- Not applicable

Which of the following statements represent your household financial situation best?

- With current incomes level we live well,
- With current incomes level we are doing fine,
- With current incomes level we hardly cope with the situation,
- With current incomes level we can't handle this situation,
- Refuse to answer

What is your nationality?

____

What country are you currently living in?

____

MEDICAL
UNIVERSITY
OF GDAŃSK

www.mug.edu.pl
58 349 11 11
info@gumed.edu.pl
ul. M. Skłodowskiej-Curie 3a,
80-210 Gdańsk

City size:

- village,
- city with under 50 K citizens,
- city with between 50K and 150K citizens,
- city with between 150K and 500K citizens,
- city with over 500K citizens

What type of building do you currently live in?

- block of flats,
- tenement house,
- detached house,
- terraced house,
- academic/room,
- other

How many people does your household include:

- 1,
- 2,
- 3,
- 4,
- 5 and more

Do You agree to participate in next steps of this research in the future? If yes, please provide your contact e-mail address. Providing an e-mail address is voluntary. Personal data administrator will be University Of Gdańsk located on Jana Bażyńskiego 8 street in Gdańsk, Poland and Gdańsk Medical University located on M.Skłodowskiej-Curie 3a street in Gdańsk, Poland. Personal information provided in this form will be used only in regards to this research.

Thank you for your time.
