# Supplementary material for: Attitudes and Perceptions of Health Protection Measures Against the Spread of COVID-19 in Italy and Poland
Source: Front Psychol. 2021 Dec 24;12:805790. doi: 10.3389/fpsyg.2021.805790 (PMC8754188; doi:10.3389/fpsyg.2021.805790)
Supplement: Supplementary file 3 [file Image_2.pdf]

uniss
UNIVERSITÀ DEGLI STUDI DI SASSARI

DIPARTIMENTO DI SCIENZE MEDICHE CHIRURGICHE E SPERIMENTALI

viale S. Pietro, 43 07100 SASSARI (Italy)
tel. +39 079 228222
p.iva e c.f. 00196350904
dip.scienze.mediche.chirurgiche.sperimentali@pec.uniss.it
www.scienzemedchirsper.uniss.it www.uniss.it

Dear respondents,

With the recent onset of the COVID-19 pandemic we are currently facing an unprecedented situation that affects our everyday life.

In this study we are asking you for your opinion on the requirements of social isolation during COVID-19 pandemic, as well as for the description on how isolation, quarantine and other disturbances influence your comfort.

Results will help to recognize how how separation from other people. We kindly request you to spend about 10 minutes answering the questtions. Every single response is valuable to us. All data collected would for use of scientific purposes only. Personal data will be confidential, coded for statistical analyses. Taking part in our query is voluntary. The only restriction is age above 18.

- Quarantine- obligatory separation of an individual suspected of being a host of an infectious disease

- Home isolation-restriction in contact or lack of direct social intercommunication (face to-face contact)
